# The 30 kb deletion in the *APOBEC3* cluster decreases *APOBEC3A* and *APOBEC3B* expression and creates a transcriptionally active hybrid gene but does not associate with breast cancer in the European population

**DOI:** 10.18632/oncotarget.19400

**Published:** 2017-07-19

**Authors:** Katarzyna Klonowska, Wojciech Kluzniak, Bogna Rusak, Anna Jakubowska, Magdalena Ratajska, Natalia Krawczynska, Danuta Vasilevska, Karol Czubak, Marzena Wojciechowska, Cezary Cybulski, Jan Lubinski, Piotr Kozlowski

**Affiliations:** ^1^ Department of Molecular Genetics, Institute of Bioorganic Chemistry, Polish Academy of Sciences, Poznan, Poland; ^2^ Department of Genetics and Pathology, International Hereditary Cancer Center, Pomeranian Medical University, Szczecin, Poland; ^3^ Department of Biology and Medical Genetics, Medical University of Gdansk, Gdansk, Poland; ^4^ Department of Gynecology, Centre of Obstetrics and Gynecology, Vilnius University Hospital Santariskiu Klinikos, Vilnius, Lithuania

**Keywords:** *APOBEC3B*, hereditary breast cancer, MLPA, copy number variation (CNV), meta-analysis

## Abstract

*APOBEC3B*, in addition to other members of the *APOBEC3* gene family, has recently been intensively studied due to its identification as a gene whose activation in cancer is responsible for a specific pattern of massively occurring somatic mutations. It was recently shown that a common large deletion in the *APOBEC3* cluster (the *APOBEC3B* deletion) may increase the risk of breast cancer. However, conflicting evidence regarding this association was also reported. In the first step of our study, using different approaches, including an in-house designed multiplex ligation-dependent probe amplification assay, we analyzed the structure of the deletion and showed that although the breakpoints are located in highly homologous regions, which may generate recurrent occurrence of similar but not identical deletions, there is no sign of deletion heterogeneity. This knowledge allowed us to distinguish transcripts of all affected genes, including the highly homologous canonical *APOBEC3A* and *APOBEC3B*, and the hybrid *APOBEC3A/APOBEC3B* gene. We unambiguously confirmed the presence of the hybrid transcript and showed that the *APOBEC3B* deletion negatively correlates with *APOBEC3A* and *APOBEC3B* expression and positively correlates with *APOBEC3A/APOBEC3B* expression, whose mRNA level is >10-fold and >1500-fold lower than the level of *APOBEC3A* and *APOBEC3B*, respectively. In the next step, we performed a large-scale association study in three different cohorts (2972 cases and 3682 controls) and showed no association of the deletion with breast cancer, familial breast cancer or ovarian cancer. Further, we conducted a meta-analysis that confirmed the lack of the association of the deletion with breast cancer in non-Asian populations.

## INTRODUCTION

Breast cancer is the primary cause of cancer-associated death among women worldwide. The probability of developing breast cancer is modulated by an interplay of lifestyle, environmental, and genetic factors. The overall heritability (h^2^) of breast cancer was estimated at approximately 30% [1]. Inherited breast cancer cases that aggregate in families constitute five to ten percent of all breast cancer cases. Highly penetrant germline mutations in *BRCA1* and *BRCA2* and in several genes associated with various hereditary cancer syndromes explain 16-40% of all breast cancer cases that aggregate in families [2–4]. Moreover, it is estimated that mutations in several susceptible genes of moderate penetrance, e.g., *ATM*, *CHEK2* or *NBN*, account for another 5% of all familial breast cancer cases [2, 5, 6]. Recently, international collaborative analyses involving genome-wide association studies (GWASs) have revealed common low-penetrance single nucleotide polymorphisms (SNPs) in 94 loci that are individually associated with breast cancer. It is assumed that the cooperative effect of the identified SNPs may underlie more than 20% of breast cancer heritability [7–9]. Overall, the genetic background of breast cancer predisposition in approximately 50% of breast cancer cases aggregated in families still remains to be explained [4, 7].

It was presumed that investigating copy number variants (CNVs) may uncover a substantial part of still unidentified genetic loci related to the susceptibility to various complex diseases [10]. CNVs have been shown to be associated with several complex diseases, including HIV infection and AIDS development [11], osteoporosis [12], Crohn's disease [13] and autism [14]. Recently, it was also suggested that CNVs may underlie hidden susceptibility to breast cancer [15–17]. One common CNV that potentially increases the risk of breast cancer is the deletion of the *APOBEC3B* gene, which occurs with a high allelic frequency in East Asian (37%), Amerindian (58%) and Oceanic populations (93%) and with a moderate (6%) or low (1%) allelic frequency in European and African populations, respectively [18]. The germline *APOBEC3B* deletion, comprising an ~30 kb genomic region, extends between the last noncoding exon of *APOBEC3A* and the eighth exon of *APOBEC3B* and leads to the complete removal of the *APOBEC3B* protein-coding region. It was suggested that as a result of the *APOBEC3B* deletion, a hybrid gene, *APOBEC3A/APOBEC3B*, which possesses a coding sequence of *APOBEC3A* (exon 1 – exon 5) and 3’ untranslated region (3’UTR) from *APOBEC3B* (exon 8), is generated. It can be assumed that transcript generated from the *APOBEC3B* deletion allele is subjected to different cellular regulation. Although the function of the two distinct APOBEC3A and APOBEC3B proteins is being intensively studied (reviewed in [19–24]), little is known about the influence of the germline *APOBEC3B* deletion on the expression of affected genes (genes overlapped by the *APOBEC3B* deletion, i.e., *APOBEC3A*, *APOBEC3B*, and the *APOBEC3A/APOBEC3B* hybrid). Therefore, direct evidence that the presumed *APOBEC3A/APOBEC3B* hybrid transcript actually arises and a detailed elucidation of its structure are of high importance. The knowledge of the exact structure of the hybrid transcript is vital for the design of a comprehensive tests for analysis of the influence of the *APOBEC3B* deletion genotype on the expression of *APOBEC3B, APOBEC3A* and the *APOBEC3A/APOBEC3B* hybrid gene, which would deepen the current knowledge of the functional consequences of the *APOBEC3B* deletion.

Several associations of the *APOBEC3B* deletion with different complex human diseases have already been reported [17, 25–42], including an association of the *APOBEC3B* deletion with breast cancer risk in a Chinese population (OR=1.31/1.76 for one/two copies of the deletion) identified by Long and coworkers [17] and in two smaller studies in two other Asiatic populations, i.e., Iranian [36] and Malaysian [40]. In contrast, association of the *APOBEC3B* deletion with breast cancer is much less conclusive in European populations. The association was confirmed by another study of the Long group in a European-American population [31] but not in a Swedish population [37]. The association was also not confirmed in two smaller studies in Indian [38] and Moroccan [39] populations. It was concluded that in the Caucasian population, the relationship of the *APOBEC3B* deletion with increased breast cancer risk cannot be convincingly stated; therefore, further large-scale comprehensive association studies are necessary [43]. The currently available results report risk related to the *APOBEC3B* deletion in groups of unselected breast cancer cases. The role of the *APOBEC3B* deletion in familial breast cancer predisposition remains to be elucidated.

The APOBEC3A and APOBEC3B proteins belong to Activation-Induced Cytidine Deaminase (AID)/ Apolipoprotein B mRNA Editing Enzyme, Catalytic Polypeptide-like (APOBEC) family, which consists of 11 cytidine deaminases. APOBEC3A and APOBEC3B possess the capability of introducing sequence alterations in single-stranded DNA (ssDNA) and are involved in various vital cellular processes (reviewed in [19, 20]), including innate immune response against retroviruses (e.g., HTLV1) [44] and DNA viruses [e.g., HBV (e.g., [32, 45, 46]) and HPV (e.g., [47, 48])], regulation of retrotransposon element movement (e.g., [49–51]), and regulation of DNA methylation (e.g., [52–54]). More recently, APOBEC3B and APOBEC3A were also reported to be mutagenic enzymes whose activation in cancer is responsible for specific patterns of massively occurring somatic mutations [55, 56], referred to as *kataegis* (from the Greek for “thunderstorm”) [57] or “mutation clusters” [58]. These patterns were observed in several cancer types, including breast cancer [59–62]. However, newer reports indicate that some controversies regarding specificities and the role of particular APOBEC3s in *kataegis* also exist [63, 64].

In this study, we determined the exact structure of the hybrid *APOBEC3A*/*APOBEC3B* gene and provided direct evidence of the presence of the hybrid *APOBEC3A*/*APOBEC3B* transcript in individuals carrying allele with the *APOBEC3B* deletion. We also analyzed the relationship between the *APOBEC3B* deletion genotypes and the expression of the affected genes, i.e., *APOBEC3A, APOBEC3B* and hybrid *APOBEC3A*/*APOBEC3B*, and showed that the *APOBEC3B* deletion negatively correlates with the expression of *APOBEC3A* and *APOBEC3B* and positively correlates with expression of *APOBEC3A/APOBEC3B*. We also performed a large-scale, case-control study of the association of the *APOBEC3B* deletion with breast and ovarian cancer in three different European cohorts (encompassing >6500 samples), which revealed the lack of association of the *APOBEC3B* deletion with breast and ovarian cancer in European populations. To obtain a more global view of the role of the *APOBEC3B* deletion in cancer predisposition, we also conducted a comprehensive meta-analysis, considering all association studies of the deletion with breast and other types of cancer.

## RESULTS

### Design of the A3Bdel_PCR assay and comprehensive analysis of the structure of the *APOBEC3B* deletion

**Figure 1:**
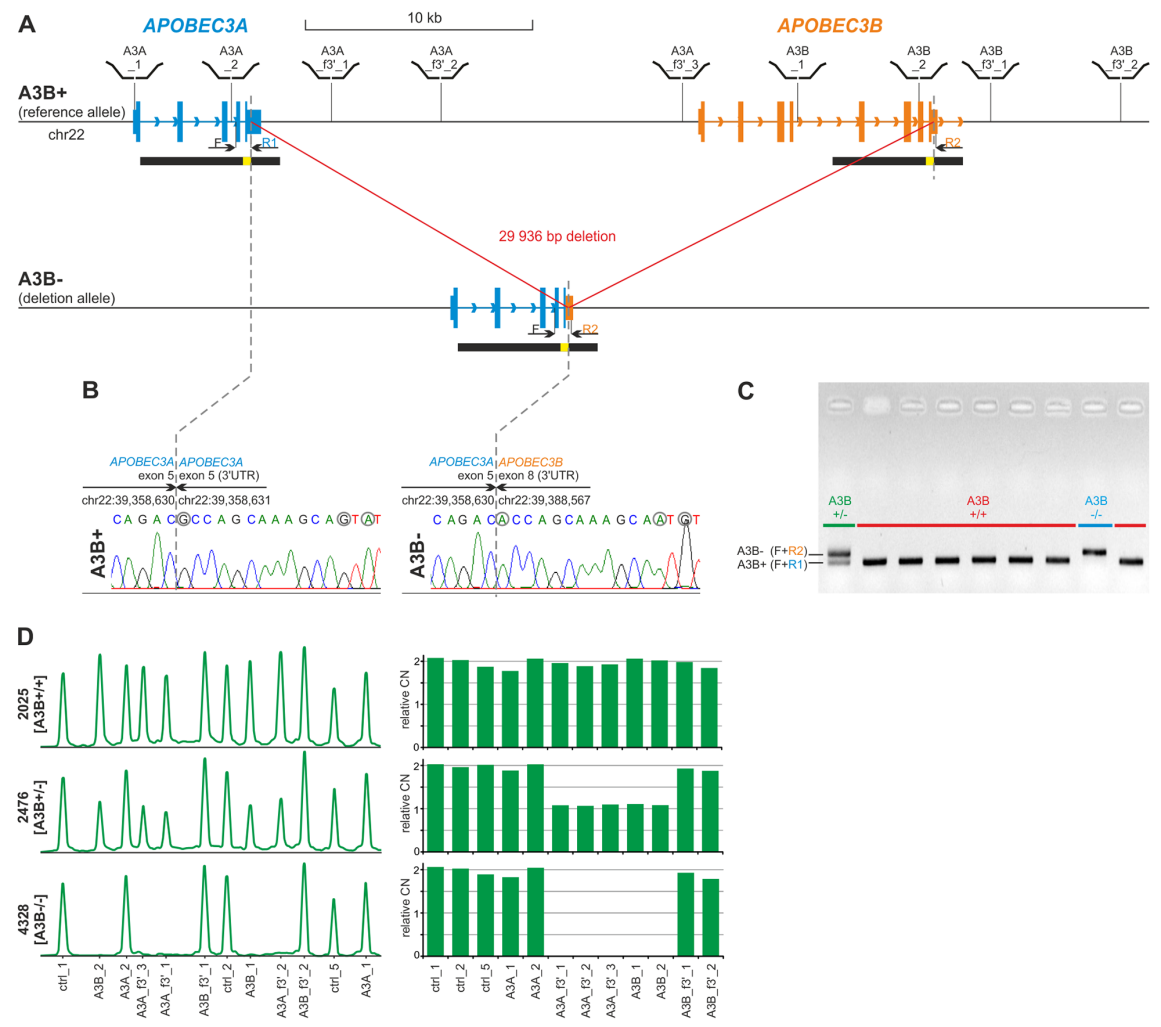
Structure of the *APOBEC3B* deletion and design of the PCR-based and MLPA-based genotyping assays **(A)** Structure of A3B+ and A3B- alleles. Blue and orange vertical rectangles indicate exons of *APOBEC3A* and *APOBEC3B*, respectively. Higher and lower rectangles indicate coding and UTR sequences, respectively. Arrowheads along intron lines indicate the direction of the genes. Black horizontal bars under the gene schemes indicate highly homologous (~95%) segmentally duplicated regions, with a yellow inset indicating 350 bp redundant fragment of 100% homology. Positions of MLPA probes used for A3Bdel_MLPA assay are schematically depicted above the region map; positions of F, R1 and R2 primers used for sequencing and in A3Bdel_PCR assay are indicated under the map. **(B)** Results of Sanger sequencing of deletion breakpoints amplified with the F-R1 (left-hand side) and F-R2 (right-hand side) pairs of primers, respectively. Dotted lines indicate exact breakpoint positions according to HGVS recommendation (after the last nucleotide of the redundant sequence). Sequencing was performed using homozygous A3B+/+ and A3B-/- samples. Nucleotides that distinguish the A3B+ and A3B- alleles are circled in gray. **(C)** Visualization (agarose gel with EtBr staining) of PCR products of the A3Bdel_PCR assay. A3B+/+ genotypes without deletion; A3B-/- genotype with homozygous deletion; A3B+/- genotype with heterozygous deletion. **(D)** Exemplary results of the A3Bdel_MLPA analysis. The left-hand side panel: the MLPA electropherograms of the representative samples with A3B+/+, A3B+/-, and A3B-/- genotypes. The probe IDs are shown under the electropherograms. The right-hand side panel: bar plots (corresponding to the electropherograms shown on left-hand side) representing the normalized copy number value (y-axis) of each probe (x-axis).

As breakpoints of the deletion overlap with extended highly homologous regions (Figure 1, segmental duplications of 95% similarity cover almost entire *APOBEC3A* and 3’-half of *APOBEC3B*), determination of their exact positions may be redundant. Therefore, in the first step we designed a simple single-tube A3Bdel_PCR assay to distinguish the reference (A3B+) and the deletion (A3B-) alleles and to unequivocally confirm the exact size and position of the deletion. The test takes advantage of nucleotide positions specific for particular duplicated regions defined based on a careful analysis of the reference sequence (hg19) and on previous results [18]. The assay consists of three PCR primers, i.e., one forward primer (F) and two distinct reverse primers (R1 and R2) (Figure 1). F is located on the border of *APOBEC3A* intron 3 and exon 4, upstream of the presumed 5’-breakpoint of the *APOBEC3B* deletion; R1 is located in the *APOBEC3A* exon 5 downstream of the presumed 5’-breakpoint of the deletion; and R2 is specific to the sequence within the *APOBEC3B* exon 8 downstream of the presumed 3’-breakpoint of the deletion (Figure 1A). R1 and R2 primers distinguish the A3B+ and A3B- alleles, respectively. The primers are localized in such a way that the amplicons corresponding to the A3B+ and the A3B- are of different lengths, which distinguish them and identify the *APOBEC3B* deletion genotypes (Figure 1). With the use of the designed A3Bdel_PCR primers, we performed a sequencing analysis that determined the deletion breakpoints at a single-nucleotide resolution (Figure 1). The sequencing analysis refined the *APOBEC3B* deletion to a 29 936 bp genomic region. It has to be noted, however, that the 5’ or 3’ breakpoints of the *APOBEC3B* deletion lie within a 350 bp sequence that is identical on both sides of the deletion; therefore, the exact position of the deletion depends on the assumed convention/nomenclature (according to the HGVS nomenclature: GRCh37/hg19: g.chr22:39358631_g.chr22:39388566del or APOBEC3A:c.717_APOBEC3B:c.1265del).

The location of the breakpoints of the deletion in almost identical, segmentally duplicated regions may induce recurrent occurrence of similar but not identical deletions arising due to non-allelic homologous re-combination (NAHR). Therefore, to exclude the potential heterogeneity of the *APOBEC3B* deletion, we utilized multiplex ligation-dependent probe amplification (MLPA), which is the method of choice for the analysis of large deletions. Because commercial MLPA assays are available only for a limited number of genes and there is no such assay for the *APOBEC3A* and *APOBEC3B* genes, we designed a homemade A3Bdel_MLPA assay. The assay was designed and generated according to a strategy previously developed in our group [65–68]. The A3Bdel_MLPA assay is composed of 12 probes, i.e., 4 probes located in close proximity to the breakpoints within flanking sequences of the deletion, 5 probes located in the presumed deletion region, and 3 control probes specific to copy-number stable regions located in chromosomes 1, 2, and 22 (for details, see Materials and Methods, Figure 1A and Supplementary Table 1). The A3Bdel_MLPA probe set was verified to provide robust, high-quality results in a series of optimization experiments performed using a set of reference gDNA samples. The optimized A3Bdel_MLPA assay was used to analyze two panels of gDNA samples, i.e., a panel of 31 samples from the HapMap project and a panel of 17 samples derived from women with breast and/or ovarian cancer with different *APOBEC3B* deletion genotypes that were previously determined with the use of the A3Bdel_PCR assay (Figure 1D and Supplementary Figure 1). We observed a perfect correlation between the obtained MLPA patterns and the *APOBEC3B* deletion genotypes identified in the A3Bdel_PCR analysis, which indicates the lack of heterogeneity in the structure of the *APOBEC3B* deletion.

### Effect of the *APOBEC3B* deletion on the expression of the affected genes

**Figure 2:**
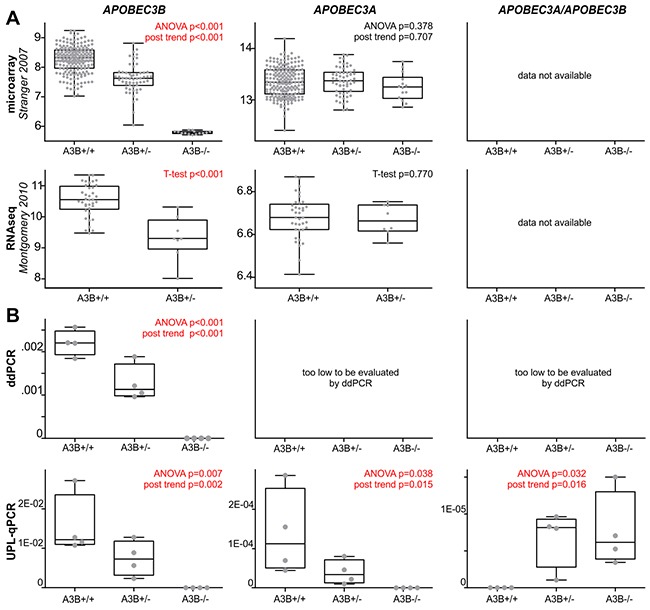
Effect of the *APOBEC3B* deletion on the expression of the affected genes, i.e., *APOBEC3B*, *APOBEC3A*, and *APOBEC3A/APOBEC3B* **(A)** Comparison of the *APOBEC3B* deletion genotypes (A3B+/+, A3B+/-, A3B-/-; x-axes) in HapMap samples with relative expression (y-axes) level of the affected genes (indicated above) retrieved from microarray (upper row, [69]) and RNAseq (lower row, [70]) whole genome mRNA profiling datasets. It has to be noted that panel of HapMap samples analyzed in RNAseq study did not comprise samples with A3B-/- genotype. **(B)** Experimental analysis of expression of the affected genes in 12 HapMap cell lines with A3B+/+ (n=4), A3B+/- (n=4) and A3B-/- (n=4). The expression analysis was performed with ddPCR (upper row) and UPL-qPCR (lower row). Note that due to very low levels of *APOBEC3A* and *APOBEC3A/APOBEC3B* expression (>>1000 lower than *APOBEC3B*), they could not be reliably evaluated with ddPCR. The box-and-whisker plots summarize the distribution of the relative expression data points determined by microarray, RNAseq, ddPCR, and UPL-qPCR. The band inside of each box represents the median, and the upper and lower edges of the box represent 1st and 3rd quartile of distribution. Whiskers indicate the lowest and the highest observed values.

In the first step, to assess the effect of the *APOBEC3B* deletion on *APOBEC3A* and *APOBEC3B* expression, we took advantage of whole genome mRNA profiling datasets regarding panels of HapMap samples derived from LCLs (lymphoblast cell lines) from B-lymphocytes. The genome-wide expression datasets were generated for 270 and 45 samples from basic (phase I/II) HapMap panel, with the use of microarray [69] and RNAseq [70] technology, respectively. From the datasets, we extracted data regarding the expression levels of *APOBEC3A* and *APOBEC3B* and compared them with the *APOBEC3B* deletion genotypes determined for these samples by [18] and independently determined by us (data not shown). As shown in Figure 2A, the *APOBEC3B* deletion genotype (presence of the deletion) negatively correlates with *APOBEC3B* expression [both microarray (R=-0.74, p<0.001) and RNAseq (R=-0.65, p<0.001) data] but not with *APOBEC3A* expression. It has to be noted, however, that lack of correlation of *APOBEC3A* expression with the deletion genotype may result from (i) very low levels of *APOBEC3A* expression (in comparison to *APOBEC3B*) in some tissues and cell subsets, including T- and B-lymphocytes and breast cancer cell lines [55, 64, 71–73], or (ii) high homology between *APOBEC3A* and *APOBEC3B* and other *APOBEC* family members that might lead to the mismapping of RNAseq reads or cross-hybridization of *APOBEC3A*-specific probes [55, 64]. The lack of consistency in the measurement of the *APOBEC3A* expression level between the studies (in [70], it is lower than the level of *APOBEC3B*, but in [69], it is higher than the level of *APOBEC3B*) strongly suggests the occurrence of the cross-hybridization.

Therefore, to further investigate the effect that the *APOBEC3B* deletion has on the expression of *APOBEC3A* and *APOBEC3B* as well as the *APOBEC3A/APOBEC3B* hybrid gene, occurring in the presence of the *APOBEC3B* deletion, we performed our own experimental analysis. First, we performed a sequencing analysis of the presumed *APOBEC3B/APOBEC3A* hybrid transcript. The analysis unequivocally confirmed that the transcript is actually generated from the allele with the *APOBEC3B* deletion and defined its structure at a single-nucleotide resolution (Supplementary Figure 2). Taking advantage of the gathered information about the precise sequence structure of the *APOBEC3B* deletion and the hybrid transcript, we designed A3A_exp, A3B_exp and A3A/A3B_exp assays for expression analysis that distinguished the hybrid *APOBEC3A/APOBEC3B* transcript from the canonical *APOBEC3A* and *APOBEC3B* transcripts (for details, see Materials and Methods). For the purpose of the analysis, we selected 12 LCLs from the HapMap project representing different *APOBEC3B* genotypes, i.e., 4 cell lines with a reference *APOBEC3B* genotype (A3B+/+; 2 *APOBEC3B* copies), 4 cell lines with a heterozygous deletion of *APOBEC3B* (A3B+/−; 1 *APOBEC3B* copy), and 4 cell lines with a homozygous deletion of *APOBEC3B* (A3B−/−; 0 *APOBEC3B* copies). The A3A_exp, A3B_exp and A3A/A3B_exp assays were utilized for the evaluation of the expression levels in all the LCLs using droplet digital PCR (ddPCR) method. The ddPCR analysis revealed a negative correlation between the *APOBEC3B* deletion genotype and the expression of the *APOBEC3B* gene. In cell lines with A3B+/+, A3B+/− and A3B−/− genotypes, *APOBEC3B* expression decreased gradually in a nearly linear manner (R=-0.96, p<0.001) (Figure 2B and Supplementary Figure 3). However, due to the very low level signal of *APOBEC3A* and *APOBEC3A/APOBEC3B*, we were not able to reliably evaluate their expression with ddPCR. Therefore, in the next step, we used a UPL-qPCR technique that has a higher dynamic range than ddPCR. With the use of UPL-qPCR, we confirmed the negative correlation between the *APOBEC3B* deletion genotype and *APOBEC3B* expression (R=-0.82, p=0.002) and revealed a negative and positive correlation between the *APOBEC3B* deletion genotype and the expression of *APOBEC3A* (R=-0.70, p=0.015) and the expression of *APOBEC3A/APOBEC3A* (R=0.67, p=0.016), respectively (Figure 2B). As shown in Figure 2B, the expression of *APOBEC3A* and *APOBEC3A/APOBEC3B* is very low and is, respectively, ~130-fold and >1500-fold lower than the expression of *APOBEC3B*. Surprisingly, our analysis revealed that *APOBEC3A/APOBEC3B* hybrid expression is substantially lower (~12-fold) than the expression of canonical *APOBEC3A*, which indicates the role of the 3’UTR in the differential regulation of these genes and that *APOBEC3A/APOBEC3B* does not compensate for the lack of *APOBEC3A* dosage.

### Analysis of the association of the *APOBEC3B* deletion with breast and ovarian cancer risk

For association analysis, we used several case-control set-ups from three different cohorts, i.e., GDANSK (523 BC-cases; 343 OC-cases; 853 controls), SZCZECIN (2009 BC-cases; 2005 controls; 615 NH-controls), and VILNIUS (97 OC-cases; 209 controls) (for details see Materials and Methods). The size of cumulative breast cancer case-control groups was estimated based on the frequency of the deletion in the European population (~11-13% - based on our preliminary results and [18]) and expected effect (OR~1.3 – estimated based on previous studies, i.e., [17, 31]) of the *APOBEC3B* deletion in order to obtain adequate statistical power (>90%) of the analysis. All samples were genotyped using a simple single tube A3Bdel_PCR assay (for details see “Design of the A3Bdel_ PCR assay and comprehensive analysis of the structure of the *APOBEC3B* deletion” and Materials and Methods). The distribution of the *APOBEC3B* deletion in control samples in all three groups was in good agreement with that expected under Hardy–Weinberg equilibrium (HWE) (p>>0.05), which indicates the high quality of the obtained genotyping results and the lack of bias in the detection of homozygous and heterozygous deletions. Logistic regression was used to derive odds ratios (ORs) and 95% confidence intervals (CIs) for the associations between the *APOBEC3B* deletion and cancer risk.

Numbers and frequencies of alleles and genotypes identified in the case and control groups are summarized in Table 1 (breast cancer association study) and Table 2 (ovarian cancer association study). In the association analyses, we focused mostly on the dominant model of inheritance (A3B+/− and A3B−/− vs. A3B+/+) (Tables 1 and 2), but we also performed association analysis assuming additive and recessive models of inheritance (Supplementary Tables 2 and 3). The latter two models have much lower statistical power due to the low frequency (≤1%) of homozygous deletions (A3B−/− genotypes).

**Table 1:** Analysis of the association of the *APOBEC3B* deletion with breast cancer risk using dominant model of inheritance (A3B+/− and A3B−/− vs. A3B+/+)

group	Genotypes	No of cases(%)	No of controls(%)	OR(95%CI)	AdjustedOR(95%CI)
GDANSKBC cases (n=523)vs. unselected controls(n=853)	A3B+/+	450 (86.04%)	759 (88.98%)	1.31(0.94-1.82)p=0.11	-
	A3B+/−	71 (13.58%)	91 (10.67%)		
	A3B−/−	2 (0.38%)	3 (0.35%)		
	A3B+/− and A3B−/−	73 (13.96%)	94 (11.02%)		
SZCZECINBC cases (n=2009)vs. unselected controls(n=2005)	A3B+/+	1764 (87.80%)	1733 (86.43%)	0.89(0.74-1.07)p=0.20	0.90(0.75-1.08)^a^p=0.26
	A3B+/−	235 (11.70%)	267 (13.32%)		
	A3B−/−	10 (0.50%)	5 (0.25%)		
	A3B+/− and A3B−/−	245 (12.20%)	272 (13.57%)		
SZCZECINBC cases (n=2009)vs. NH-controls (n=615)	A3B+/+	1764 (87.80%)	542 (88.13%)	1.03(0.78-1.36)p=0.83	0.95(0.70-1.29)^a^p=0.73
	A3B+/−	235 (11.70%)	68 (11.06%)		
	A3B−/−	10 (0.50%)	5 (0.81%)		
	A3B+/− and A3B−/−	245 (12.20%)	73 (11.87%)		
GDANSK+SZCZECINBC cases (n=2532)vs. unselected controls(n=2858)	A3B+/+	2214 (87.44%)	2492 (87.19%)	0.98(0.83-1.15)p=0.79	0.97(0.83-1.14)^b^p=0.73
	A3B+/−	306 (12.09%)	358 (12.53%)		
	A3B−/−	12 (0.47%)	8 (0.28%)		
	A3B+/− and A3B−/−	318 (12.56%)	366 (12.81%)		
GDANSK+SZCZECINfamilial BC cases (n=640)vs. unselected controls(n=2858)	A3B+/+	554 (86.56%)	2492 (87.19%)	1.06(0.82-1.36)p=0.67	1.15(0.87-1.52)^b^p=0.32
	A3B+/−	84 (13.13%)	358 (12.53%)		
	A3B−/−	2 (0.31%)	8 (0.28%)		
	A3B+/− and A3B−/−	86 (13.44%)	366 (12.81%)		

^a^Adjusted for age; ^b^adjusted for the origin of the study.

**Table 2:** Analysis of the association of the *APOBEC3B* deletion with ovarian cancer risk using dominant model of inheritance (A3B+/− and A3B−/− vs. A3B+/+)

Group	Genotypes	No of cases(%)	No of controls(%)	OR(95%CI)	AdjustedOR(95%CI)
GDANSK: OCcases (n=343)vs. unselected controls(n=853)	A3B+/+	313 (91.25%)	759 (88.98%)	0.77(0.50-1.19)p=0.24	-
	A3B+/−	28 (8.16%)	91 (10.67%)		
	A3B−/−	2 (0.58%)	3 (0.35%)		
	A3B+/− and A3B−/−	30 (8.75%)	94 (11.02%)		
VILNIUS: OCcases (n=97)vs. unselected controls(n=209)	A3B+/+	90 (92.78%)	187 (89.47%)	0.66(0.27-1.61)p=0.36	-
	A3B+/−	6 (6.19%)	22 (10.53%)		
	A3B−/−	1 (1.03%)	0 (0%)		
	A3B+/− and A3B−/−	7 (7.22%)	22 (10.53%)		
GDANSK + VILNIUS: OCcases (n=440)vs. unselected controls(n=1062)	A3B+/+	403 (91.59%)	946 (89.08%)	0.75(0.51-1.10)p=0.14	0.75(0.51-1.11)^b^p=0.15
	A3B+/−	34 (7.73%)	113 (10.64%)		
	A3B−/−	3 (0.68%)	3 (0.28%)		
	A3B+/− and A3B−/−	37 (8.41%)	116 (10.92%)		

^b^Adjusted for the origin of the study.

As shown in Table 1, the frequency of the *APOBEC3B* deletion in breast cancer cases does not significantly differ from that observed in population controls in GDANSK[OR(95%CI)=1.31(0.94-1.82), p=0.11)] or SZCZECIN [OR(95%CI)=0.89(0.74-1.07), p=0.20]. It also does not differ from cancer-free controls (SZCZECIN-NH controls; negative for any cancer and negative for family history of cancer) [OR(95%CI)=1.03(0.78-1.36), p=0.83]. To increase the power of the analysis, we combined SZCZECIN and GDANSK cohorts but observed no association [OR(95%CI)=0.98(0.83-1.15), p=0.79]. Adjusting for the origin of the samples did not significantly influence the results. We also did not see an association of the *APOBEC3B* deletion with familial breast cancer cases, which were selected from the GDANSK and SZCZECIN breast cancer cases [OR (95%CI)=1.06(0.82-1.36), p=0.67].

The *APOBEC3B* deletion also does not show association with ovarian cancer in either GDANSK [OR(95%CI)=0.77(0.50-1.19), p=0.24] or VILNIUS [OR(95%CI)=0.66(0.27-1.61), p=0.36]. It also does not associate with ovarian cancer in a combined GDANSK/VILNIUS cohort [OR(95%CI)=0.75(0.51-1.10), p=0.14]. Adjusting for the origin of the samples did not significantly influence the results.

### Meta-analysis of association studies of the *APOBEC3B* deletion with cancer

**Figure 3:**
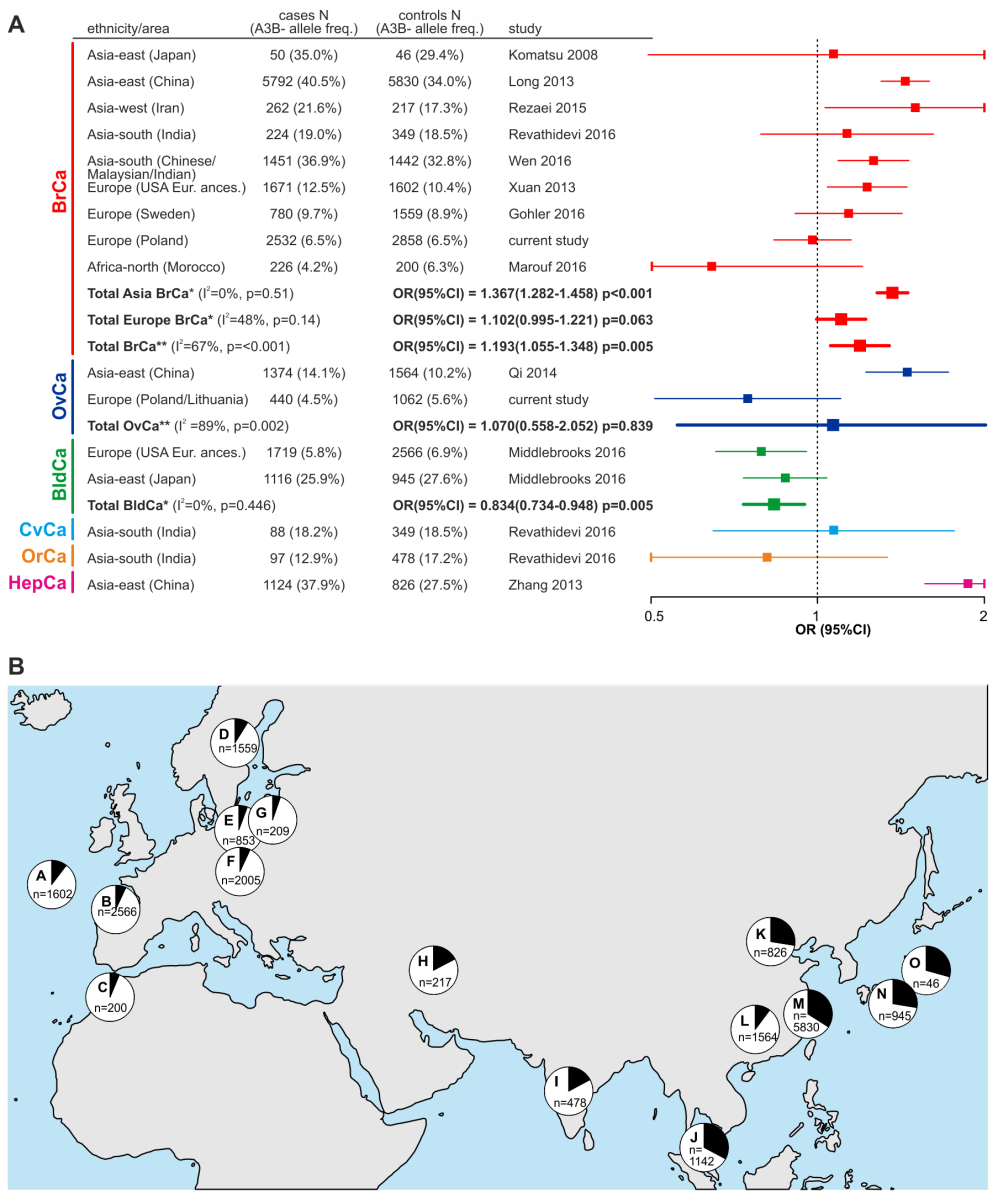
Meta-analysis of association studies of the *APOBEC3B* deletion with breast and other types of cancer **(A)** Forest plot summarizing results of the meta-analysis. Characteristics of the studies used in the meta-analysis are shown on the left side of the plot. The plot illustrates the measure of the effect of the *APOBEC3B* deletion on predisposition to particular cancer types, i.e., OR (square) with the corresponding 95% CI (horizontal lines), observed in different studies enrolled in the meta-analysis. The meta-analysis was performed under the dominant model of inheritance (A3B+/+ and A3B+/- vs. A3B-/-). Weighted odds ratios with the corresponding 95%Cis (squares with horizontal lines in bold) were obtained using Mantel-Haenszel method under fixed* or random** effects models, depending on results of heterogeneity tests (p>0.10 for the Q test and I<50% were considered to indicate a lack of significant heterogeneity). The vertical dotted line indicates no effect (OR=1) of the *APOBEC3B* deletion on cancer predisposition. The OR values are plotted on a logarithmic scale to obtain symmetrical CIs and equivalent visualization of ORs of values greater and lower than 1. Horizontal lines corresponding to CIs that are out of range of 0.5-2 have been cut (vertical line) for visualization purposes. BrCa - breast cancer; OvCa - ovarian cancer; BldCa - bladder cancer; CvCa - cervical cancer; OrCa - oral cancer; HepCa - hepatocellular cancer. **(B)** Worldwide distribution of the allelic frequencies (black segments in pie charts) of the *APOBEC3B* deletion in control groups used in studies that were enrolled in the meta-analysis. Each letter indicates particular geographic region/population from a particular study, i.e., A - USA European ancestry [31]; B - Spain/USA European ancestry [41]; C - Morocco [39]; D - Sweden [37]; E - Poland (GDANSK) (current study); F - Poland (SZCZECIN) (current study); G - Lithuania (VILNIUS) (current study); H - Iran [36]; I - India [38]; J - Malaysia [40]; K - China [32]; L - China [34]; M - China [17]; N - Japan [41]; O - Japan [25]. “n” indicates the number of samples in the control groups in each study.

The association of the *APOBEC3B* deletion with cancer has been analyzed within eleven case-control studies conducted in populations of different ethnicities, different cancer types and different sizes [published and available in PubMed up to February 2017 [17, 25, 31, 32, 34, 36–41]. It has to be noted that the results obtained in the analyses are inconsistent and even conflicting. To obtain a more global view on the effect of the *APOBEC3B* deletion on cancer, we conducted a comprehensive meta-analysis that considered all case-control studies of the deletion (including our own) performed using cases with different cancer types (predominantly breast cancer). In total, 17637 cases and 19387 controls were enrolled in our meta-analysis (not including single studies of the deletion association with cervical, oral [38] and hepatocellular [32] cancer) (Figure 3A). As shown in Figure 3A, our meta-analysis revealed the consistent association of the *APOBEC3B* deletion with breast cancer in Asian populations [OR(95%CI)=1.367(1.282-1.458), p<0.001], but in European populations, the effect of the *APOBEC3B* deletion was much smaller and not significant [OR(95%CI)=1.102(0.995-1.221), p=0.063]. A single study of a north African population also did not show an association of the *APOBEC3B* deletion with breast cancer. Consequently, meta-analysis in the general population showed significant but modest association of the *APOBEC3B* deletion with breast cancer [OR(95%CI)=1.193(1.055-1.348), p=0.005]. Additionally, our meta-analysis revealed the lack of the association of the *APOBEC3B* deletion with ovarian cancer [OR(95%CI)=1.070(0.558-2.052), p=0.839] and opposite association (protective effect) of the deletion with bladder cancer [OR(95%CI)=0.834(0.734-0.948), p=0.005]. It has to be noted, however, that these latter two meta-analyses have much lower statistical power (each composed of only two studies). Additionally, Middlebrooks and colleagues [41] showed that the effect of the *APOBEC3B* deletion on bladder cancer is mostly driven by SNP rs1014971 [being in linkage disequilibrium (LD) with the deletion], and mostly disappears after adjustment for this SNP genotype [OR(95%CI)=0.88(0.72-1.07), p=0.21 and 0.96(0.79-1.16), p=0.67 in European and Japanese populations, respectively]. In the forest plot summarizing the *APOBEC3B* deletion association studies (Figure 3A), we also included the results of Revathidevi and colleagues [38] and Zhang and colleagues [32], which were only studies of cervical/oral and HBV-related hepatocellular cancer, respectively, and were therefore not included in the meta-analysis. The frequency of the deletion allele varies significantly across different ethnic groups/geographic regions. Drastic differences in the *APOBEC3B* deletion frequency among populations suggest that it is subjected to different selective pressures in human populations, it is functionally important, and it possesses the potential to modify phenotypes. It transpires that the worldwide distribution of the deletion frequency among control groups from studies enrolled in our meta-analysis resembles the distribution previously determined by Kidd and colleagues [18], with a mean frequency of 28.5% in Asia and 7.8% in Europe (worldwide frequency: 19.33%), which indicates that the studies enrolled in our meta-analysis are reliable and devoid of evident genotyping errors/biases (Figure 3B).

## DISCUSSION

The two major reasons for the recent significant intensification of efforts on understanding the functional importance of the *APOBEC3B* gene are (i) the observation that activation of *APOBEC3B* in cancer leads to the generation of specific hypermutation signatures in breast and other cancer genomes [55, 57, 58] and (ii) the association of the *APOBEC3B* gene deletion with breast cancer risk [17, 25].

The aim of our study was to extend the current knowledge of the structure of the *APOBEC3B* deletion, its influence on the expression of the affected genes, and its association with breast and ovarian cancer predisposition in the European population. We determined the exact structure/breakpoints of the *APOBEC3B* deletion (1 nt resolution) and showed that although the breakpoints are located in highly homologous regions that may induce NAHR ([74, 75] and references within) and trigger recurrent occurrence of similar but not identical deletions, there is no sign of deletion heterogeneity. Even a small heterogeneity in the breakpoint positions would prevent the A3Bdel_PCR test from detecting the deletion and would cause discordance between A3Bdel_PCR and A3Bdel_MLPA results (not observed in our study in the panel of HapMap samples from European, African and Asiatic populations). This result strongly suggests that the deletion occurred in a single event (most likely in Africa) and then spread with the migration throughout the world, becoming common in European and Asiatic/Oceanic populations. Similar conclusions could not be derived from haplotype analysis due to low LD of flanking SNPs with the deletion [18]. We also delivered evidence of the presence of the hybrid *APOBEC3A*/*APOBEC3B* transcript in individuals carrying allele with the *APOBEC3B* deletion and confirmed its suggested structure [18, 27] with 1 nt resolution. These findings allowed us to distinguish canonical *APOBEC3A* and *APOBEC3B* genes/transcripts from the hybrid *APOBEC3A/APOBEC3B* and develop several molecular assays, i.e., A3Bdel_PCR, A3Bdel_MLPA, and A3A_exp, A3B_exp and A3A/A3B_exp, which the scientific community can use in further analyses of the complex genomic region encompassing the *APOBEC3B* deletion. It has to be noted that assays A3A_exp, A3B_exp and A3A/A3B_exp allow for an efficient distinction of the highly homologous canonical (*APOBEC3A* and *APOBEC3B*) and hybrid *APOBEC3A/APOBEC3B* transcripts and constitute an important extension of the available qPCR assays (either UPL- or TaqMan/SYBR Green-based) [55, 64, 72, 76–79] dedicated only for the analysis of canonical transcripts. E.g., in contrast to the previously developed assays, our A3A/A3B_exp assay allows to detect *APOBEC3A/APOBEC3B* transcript not only if present alone in samples with the homozygous A3B−/− deletion (e.g., [64]) but also if present on background of the other genotypes. The accuracy of transcript distinguishing of the A3A_exp, A3B_exp, and A3A/A3B_exp assays was validated using sequence analysis of PCR products (e.g., Supplementary Figure 2, lower panel). The lack of signal of the A3A_exp and A3B_exp assays in samples with homozygous deletion and lack of signal of the A3A/A3B_exp assay in samples without deletion additionally confirm the specificity of the developed assays and unequivocally show that the assays allow distinguishing the canonical and hybrid transcripts. Additionally, the developed expression assays are free of missmapping/cross-hybridization effects, affecting previously reported microarray- and RNAseq-based results and hampering distinguishing the highly homologous *APOBEC3A* and *APOBEC3B* transcripts (see also Figure 2A). This problem was reported and described with more details previously [64]. The proposed assays are cost effective [several cents (PCR genotyping) to ~5$ (MLPA) per sample)], provide reliable results and can be utilized in large-scale association and functional studies of the *APOBEC3B* deletion.

In our study, we showed for the first time the association of the *APOBEC3B* deletion with the expression of all affected genes, including the *APOBEC3A/APOBEC3B* hybrid. We performed expression analysis using the ddPCR and UPL-qPCR methods in the lymphoblastoid cell lines (in total: 12) with the naturally occurring genotypes in a natural genetic background. Additionally, we supported our results with data extracted from genome-wide datasets. Our analysis showed that the *APOBEC3B* deletion negatively correlates with the expression of *APOBEC3A* and *APOBEC3B* and positively correlates with the expression of *APOBEC3A/APOBEC3B*. Additionally, it showed that the *APOBEC3B* expression level is much higher (>100x) than the expression of *APOBEC3A*, which is still higher (>10x) than the expression of the *APOBEC3A/APOBEC3B* hybrid. Much higher expression of the canonical *APOBEC3A* than of the *APOBEC3A/APOBEC3B* hybrid gene indicates that the loss of *APOBEC3A* expression that goes with the deletion is not compensated by the increased level of the hybrid. As *APOBEC3A* and *APOBEC3A/APOBEC3B* transcripts differ only by their 3’UTRs, it strongly supports the role of the 3’UTR in differential regulation of these two sister genes, suggesting the loss of a positive regulatory element or acquisition of a negative regulatory element (decreasing transcription efficiency or stability of hybrid transcript) specific for the 3’UTR of *APOBEC3B*. This result clearly contrasts with the results of Caval et al. [73] who with the use of artificially created model genes (constructed in plasmids and transfected into human cells) demonstrated increased expression of the transcript with the *APOBEC3B* 3’UTR. The discordance between our results and the results of Caval et al. [73] is most likely a consequence of the difference between natural and plasmid-based expression system and/or different types of cell lines used in the experiments. On the other hand, our expression results are generally inline with the results of Starrett et al. [64] who, also using artificial model (CRISPR/Cas9 generated deletion), showed high expression of *APOBEC3B* and very low but similar expression of *APOBEC3A* and *APOBEC3A/APOBEC3B.* The previous studies of the *APOBEC3B* deletion genotype-expression relationship (i) did not distinguish the canonical *APOBEC3A* and the hybrid *APOBEC3A/APOBEC3B* transcripts, and/or (ii) were conducted using artificially created cell line models with transfected reporter constructs, and/or (iii) were performed with the use of single tissue or cell line samples, and/or (iv) were often inconclusive, at least partially due to high homology between *APOBEC3A* and *APOBEC3B*, as well as other members of the *APOBEC3* gene family [32, 33, 40, 63, 64, 73, 78, 80, 81].

Unequivocal confirmation of the presence of hybrid transcript (generated from A3B- allele) may strengthen the notion/hypothesis that the APOBEC3A enzyme generated from the *APOBEC3A/APOBEC3B* hybrid transcript may play an important role in the induction of *kataegis* [73]. This notion is inline with observations showing that amount of somatic mutations occurring in APOBEC3-specific sequence context is on average higher in cancer samples with the homozygous *APOBEC3B* deletion [63, 64]. On the other hand, the very low expression level of *APOBEC3A* and even lower expression of the *APOBEC3A/APOBEC3B* hybrid argues against this hypothesis and may suggest that some other member of the APOBEC3 family may play the role in generation of the APOBEC3-dependent somatic mutations. Recently, it was shown that such a gene may be one of the several variants (haplotypes) of *APOBEC3H* (i.e., *APOBEC3H-I*), which occurrence across human population additionally correlates with the occurrence of the *APOBEC3B* deletion [64]. It has to be noted, however, that the above part of the discussion is very speculative and based on often conflicting pieces of evidence reported in different studies. It indicates that much more has to be done to explain the role of the *APOBEC3* genes and the *APOBEC3B* deletion in the induction of somatic mutations in cancer.

In the next step, we performed a large-scale association study of the *APOBEC3B* deletion with breast and/or ovarian risk, which encompassed either separate or appropriately combined analyses of three European cohorts, i.e., GDANSK, SZCZECIN and VILNIUS. Our association study comprised 2972 cases and 3682 controls; it was the largest *APOBEC3B* deletion association study performed in European populations and the first *APOBEC3B* deletion association study to use the familial form of breast cancer. Our analysis revealed the lack of association of the *APOBEC3B* deletion with breast cancer risk and additionally did not show association of the deletion with familial breast cancer risk. As familial breast cancer represents a more extreme phenotype, one could expect it to show a stronger effect of the association. Therefore, the lack of association of the *APOBEC3B* deletion with familial breast cancer additionally confirms no effect of the deletion in the European population. Similarly, we also show the lack of the association of the deletion with ovarian cancer. To obtain a more global view of the relationship between the *APOBEC3B* deletion and cancer predisposition, we performed a comprehensive multilayer meta-analysis of all available studies on the association of the *APOBEC3B* deletion with cancer predisposition conducted in different populations and geographic regions. Although the meta-analysis showed substantial heterogeneity in the results obtained by different groups and a moderate global effect of the *APOBEC3B* deletion, it revealed a consistent association of the *APOBEC3B* deletion with breast cancer in Asian populations but a lack of this association in European populations. It has to be noted, however, that the association in Asian populations is driven mostly by a large seminal study [17]. The general overlap of the geographical distribution of the *APOBEC3B* deletion frequency observed in the meta-analyzed association studies with that observed before [18] exclude substantial genotyping inaccuracy in the association studies as the main source of the discordances of the *APOBEC3B* deletion effect in Asiatic and European populations. Therefore, we believe that the main source of the discordances may be differences in genetic background, such as the presence of some other causative genetic variants that in Asiatic populations share the haplotype with the *APOBEC3B* deletion but are absent or not in LD with the deletion in European populations. Some support for this hypothesis may be the analysis of HapMap Phase I SNPs that revealed different pattern of LD in regions flanking the deletion in European and Asiatic populations [18]. This analysis showed SNPs in regions directly adjacent to the deletion being in moderate LD with the deletion in Asiatic but not in European populations. Additionally, analysis of the Northern European population showed a lack of SNPs in strong LD with the deletion [37]. Later, analysis of 1000 Genomes Project data identified only one surrogate SNP, i.e., rs12628403, that showed complete LD with the deletion in a European population, lower LD in a Chinese population, and very low LD in an African population [17, 41]. Additionally, the presence of interfering environmental factors specific to particular geographic regions/populations cannot be excluded. The observed drastic differences of the frequency of the *APOBEC3B* deletion in different populations strongly support the notion of an interaction between environmentally driven selective pressure and the deletion. The function of APOBEC3B that may play a role in selective pressure may be its potential involvement in response against viral infections, e.g., HBV or HTLV1 infection [32, 44–46], or its suggested role in innate immunity against malaria [29]. However, there are some controversies and a lack of consensus on the involvement of APOBEC3B in the restriction of particular types of viruses, e.g., HIV1 (e.g., [26, 28, 33, 42]; reviewed in [20]).

Our study is not free of limitations. Our association analysis involved relatively small numbers of samples from women with ovarian cancer, which limits its power to detect potential associations with ovarian cancer risk. The performed meta-analysis is very heterogeneous in terms of proband ethnicity, geographical regions, cancer types and cancer characteristics. Additionally, some ethnic groups or geographical regions are not represented at all; e.g., there is only one small study of an African population (including no study of African-Americans) and no study of South American populations. It limits the power of some more specific (e.g., ethnic-specific) conclusions. Some studies included in the meta-analysis are very small (<<500 samples) with limited statistical power. Moreover, our expression analyses were performed with the use of only one type of cell lines. We cannot exclude that the *APOBEC3B* deletion effect may be different in different cell lines or tissues.

In conclusion, in this study, we determined the exact breakpoints of the *APOBEC3B* deletion (1 nt resolution) and confirmed its homogeneity. Additionally, we provided direct evidence for the generation of the transcriptionally active hybrid gene *APOBEC3A/APOBEC3B* from the allele with the *APOBEC3B* deletion and confirmed the suggested structure of *APOBEC3A/APOBEC3B*, which allowed us to distinguish *APOBEC3A*, *APOBEC3B*, and *APOBEC3A/APOBEC3B* expression levels. For the first time, we showed the association of the *APOBEC3B* deletion with the expression of all affected genes, including the *APOBEC3A/APOBEC3B* hybrid. We observed that the expression of *APOBEC3A/APOBEC3B* is ~10x lower than the expression of *APOBEC3A*, which implies the role of the 3’UTR in the differential regulation of these two genes coding for the same protein. We showed a lack of association of the *APOBEC3B* deletion with breast and/or ovarian risk (for the first time including familial breast cancer), which was independently validated in three European cohorts (in total: 2972 cases and 3682 controls). We also performed a comprehensive summary/visualization of all available reports on the association of the *APOBEC3B* deletion with cancer predisposition, which was obtained in our meta-analysis of association studies performed in various populations and geographic regions. It also has to be noted that within our study, we developed a variety of molecular assays that can be used for further analysis of the complex genomic region encompassing the *APOBEC3B* deletion.

## MATERIALS AND METHODS

### DNA samples

To compare the *APOBEC3B* deletion genotype with the expression of the affected genes, we used 270 reference DNA samples from the basic (phaseI/II) HapMap panel, including (i) 90 African samples from the Yoruba in Ibadan, Nigeria (YRI); (ii) 90 samples from individuals from Utah, USA, from the Centre d’Etude du Polymorphisme Humain collection (CEU); (iii) 45 samples from Han Chinese in Beijing, China (CHB); (iv) 45 samples from Japanese in Tokyo, Japan (JPT). All DNA samples were purchased from Coriell Institute for Medical Research (NJ, USA).

The *APOBEC3B* deletion association study was performed using genomic DNA samples from case-control groups collected at the Medical University of Gdansk (GDANSK group), at the International Hereditary Cancer Center in Szczecin (SZCZECIN group) and at Vilnius University Hospital Santariskiu Klinikos, Lithuania (VILNIUS group). All subjects from GDANSK, SZCZECIN, and VILNIUS provided informed written consent, and the study was approved by the appropriate local ethic committees. All subjects were Caucasians of European ancestry. Subjects from GDANSK and SZCZECIN were ethnically Poles. Subjects from VILNIUS were of mixed ethnicity, composed mostly of Lithuanians (~60%) but also Poles and Russians, with similar fractions of the ethnicities in case and control groups. In all groups, the control samples were derived from the same geographical region as the case samples.

The GDANSK group comprised 523 BC-cases [women with breast cancer from families with breast and/or ovarian cancer aggregation, as defined previously [82], negative for the 5 most common *BRCA1* mutations in the Polish population, c.68_69delAG, c.181T> G, c.3700_3704del5, c.4035delA, c.5266dupC [83, 84], 343 OC-cases (women with ovarian cancer unselected in terms of the familial history of the disease), and 853 controls (unselected population control samples).

The SZCZECIN group comprised 2009 BC-cases (women with breast cancer, unselected for familial history of the disease) and two distinct sets of control samples: 2005 SZCZECIN controls (cancer-free unselected women) and 615 SZCZECIN NH-controls (cancer-free women with a negative family history of cancer). Histological subtypes were determined for 1397 SZCZECIN BC-cases, of which 1117 (79.96%) were invasive *ductal carcinoma*, 128 (9.16%) were invasive *lobular carcinoma*, 47 (3.36%) were *ductal carcinoma* in situ, 24 (1.72%) were *carcinoma medullare*, 22 (1.57%) were *carcinoma mucinosum*, and 1-17 (0.07-1.22%) were of other subtypes of breast cancer. Breast cancer grade was determined for 1047 SZCZECIN BC-cases, of which 737 (70.39%) and 310 (29.61%) were classified as G1/G2 and G3, respectively. Status of estrogen receptor (ER) was determined for 1372 SZCZECIN BC-cases, of which 416 (30.32%) and 956 (69.68%) were ER negative and ER positive, respectively. Status of progesterone receptor (PgR) was determined for 1365 SZCZECIN BC-cases, of which 450 (32.97%) and 915 (67.03%) were PgR negative and PgR positive (PgR+), respectively. Status of human epidermal growth factor receptor 2 (HER2) was determined for 1291 SZCZECIN BC-cases, of which 1037 (80.33%) and 254 (19.67%) were HER2 negative and HER2 positive, respectively. Triple-negative (TN) status of breast cancer was determined for 218 SZCZECIN BC-cases (17.30% of cases in which all three receptors were tested). Information about familial history of cancer was recorded for 1590 cases, of which 117 (7.36%) were classified as familial breast cancer cases (according to criteria previously defined in [85]). The mean age of breast cancer diagnosis in SZCZECIN BC-cases was 54 years (range 21-92), with 187 (9.31%), 712 (35.44%) and 1110 (55.25%) women diagnosed before age 40, between the ages 40 and 50, and after age 50, respectively. A total of 52 (2.6%) SZCZECIN BC-cases were positive for one of three major *BRCA1* founder mutations in the Polish population, i.e., c.181T>G, c.4035delA, and c.5266dupC. SZCZECIN controls included women with a mean age of 52 years (range 17-94), and SZCZECIN NH-controls included women with a mean age of 67 years (range 36-95).

The VILNIUS group comprised 97 OC-cases (women with unselected ovarian cancer) and 209 controls (samples from unselected control population). Mean age of ovarian cancer diagnosis in VILNIUS OC-cases was 53 years (range 18-78). VILNIUS controls comprised women with mean age of 44 years (range 18-85).

### Analysis of the structure of the *APOBEC3B* deletion

Sequencing analyses were performed using ABI Prism 3130XL apparatus (Applied Biosystems, Carlsbad, CA, USA). The obtained sequences were analyzed with the use of Finch TV (v.1.4.0) (Geospiza Inc.).

MLPA analysis was performed using the in-house designed A3Bdel_MLPA assay. The probe-set layout was designed according to a previously proposed [65, 66] and well-validated strategy (e.g., [67, 86–88]). This strategy exclusively utilized short oligonucleotide probes that can easily be generated via standard chemical synthesis. Each probe consists of two half-probes of equal size, and the total probe length ranges from 93 to 128 nt. The target sequences for the probes were selected to avoid single nucleotide polymorphisms (SNPs), insertions/deletions, and sequences of extremely low or high guanosine-cytosine (GC) content. The sequences and detailed characteristics of all probes are shown in Supplementary Table 1. The MLPA probes were synthesized by IDT (Skokie, IL, USA). The MLPA reactions were run according to the manufacturer's general recommendations and previously published [65, 89], with reagents purchased from MRC-Holland, Amsterdam, The Netherlands. The products of the MLPA reactions were diluted 20× in HiDi formamide containing GS Liz600, which was used as a DNA sizing standard, and separated by size with capillary electrophoresis (POP7 polymer; ABI Prism 3130XL apparatus; Applied Biosystems, Carlsbad, CA, USA). The obtained electropherograms were analyzed using GeneMarker software (version 2.2.0; SoftGenetics, State College, PA, USA). The normalized signal of each probe (peak height divided by the average peak height of the control probes) was divided by the corresponding signal of a reference probe and multiplied by 2. The obtained values that correspond to the copy number of particular regions were visualized in bar graphs.

### Analysis of the *APOBEC3B* deletion genotype–expression relationship

Publically available genome-wide mRNA profiling data of different panels of HapMap samples that were used in our study are deposited at Gene Expression Omnibus (GEO) (http://www.ncbi.nlm.nih.gov/geo) database (series accession number: GSE6536), and Array Express database (https://www.ebi.ac.uk/arrayexpress/; accession number: E-MTAB-197 and E-MTAB-198) as well as at http://jungle.unige.ch/rnaseq_CEU60/ (file with normalized data “RNASEQ60_array_rep_expr.txt.gz”). The *APOBEC3B* deletion genotypes of the analyzed HapMap samples were determined before [18] and independently confirmed in our study.

Human lymphoblastoid cell lines (LCLs) GM18532, GM18537, GM18540, GM18542, GM18561, GM18570, GM18572, GM18573, GM18577, GM18579, GM18603, and GM18612 from the HapMap panel were purchased from the Coriell Institute for Medical Research (USA; http://www.coriell.org). All cell lines were cultured in Dulbecco's Modified Eagle Medium (DMEM; Lonza, Basel, Switzerland) supplemented with 10% fetal bovine serum (FBS; Sigma-Aldrich, St. Louis, MO, USA), 1% Glutamax (Cellgro, Mediatech Inc., Manassas, VA, USA), 1x antibiotic/antimycotic solution (Sigma-Aldrich), and 1x MEM non-essential amino acid solution (Sigma-Aldrich). Mycoplasma infection in cell cultures was controlled with the use of the MycoFluor™ Mycoplasma Detection Kit (Invitrogen, Carlsbad, CA, USA) according to the manufacturer's protocol.

Total RNA was extracted from cells with TRIzol (Invitrogen) according to the manufacturer's instructions. Equal amounts of RNA (2 μg) were reverse-transcribed with random primers (Invitrogen) using SuperScript III Reverse Transcriptase (Invitrogen) according to the manufacturer's protocol.

ddPCR was performed according to the manufacturer's general recommendations (Bio-Rad, CA, USA). ddPCR reactions (containing 1× EvaGreen Supermix, 200 nM primers, one-twentieth of cDNA to a final volume of 20 μL) were mixed with seventy microliters of droplet generation oil and used to form droplets in a QX200 droplet generator. The partitioned emulsion was then slowly transferred to a 96-well PCR plate (Eppendorf, Hamburg, Germany). After being heat-sealed with foil, the plates containing the droplets were PCR cycled to final point under conditions at 95°C for 5 min, then 95°C for 30 s, and 56°C for 30 s, and 72°C for 45 s for 40 cycles, 72°C for 2 min, 4°C for 5 min, 90°C for 5 min, then held at 12°C. Following PCR, samples were read on a Bio-Rad QX200 reader and data were analyzed using Quantasoft software v.1.7.4.0917 (Bio-Rad, CA, USA). Primers used in the ddPCR analyses were designed at the exon-exon junctions or in different, adjacent exons spanning a long intron to avoid amplification of the potential gDNA traces. For each analyzed cDNA sample, the following sets of PCR primers were used: (i) A3B_exp, i.e., test amplicon for *APOBEC3B*: Fwd primer 5’GACCTACGATGAGTTTGAGT3’, Rev primer 5’TTAGAGACTGAGGCCCAT3’ (amplicon length: 163 bp); (ii) A3A_exp, i.e., test amplicon for *APOBEC3A*: Fwd primer 5’CATTCTCCAGAATCAGGG3’, Rev primer 5’CTTGATCGGGAGCATAC 3’ (amplicon length: 170 bp); (iii) A3A/A3B_exp, i.e., test amplicon for *APOBEC3A/APOBEC3B* hybrid: Fwd primer 5’TGACCTACGATGAATTTAAGC3’, Rev primer 5’ATCTACTTGATCAGGAGCAC3’ (amplicon length: 293 bp), (iv) reference amplicon for *GAPDH*: Fwd primer 5’CACCACCAACTGCTTAGC3’, Rev primer 5’CATGGACTGTGGTCATGAG3’ (amplicon length: 87 bp).

UPL-qPCR analyses were performed with the use of LightCycler 480 system with probes from the Universal Probe Library (UPL) (Roche Applied Science, Penzberg, Germany), according to the manufacturer's protocol. For test amplicons, the same primer sets were used as in the ddPCR analyses. The following set of PCR primers was used as a reference amplicon for *ACTB*: Fwd primer 5’CCAACCGCGAGAAGATGA3’, Rev primer 5’CCAGAGGCGTACAGGGATAG3’ (amplicon length: 97 bp). The applied primers-UPL probes combinations were as follows: (i) *APOBEC3B* test amplicon, probe #11; (ii) *APOBEC3A* test amplicon, probe #13; (iii) *APOBEC3A/APOBEC3B* hybrid test amplicon, probe #13; (iv) *ACTB* reference amplicon, probe #64. qPCR assays were run in triplicates for 45 cycles and normalized tested gene expression level was calculated by the formula: 2^-ΔCt^, where ΔCt= Ct_test amplicon_-Ct_reference amplicon_, using the LightCycler 480 instrument software.

### A3Bdel_PCR assay for genotyping of the *APOBEC3B* deletion

Genotyping of the *APOBEC3B* deletion was performed with the use of A3Bdel_PCR assay that consists of three PCR primers, i.e., one forward primer F: 5’CCTGTCCCTTTTCAGAATTTAAGC3’, and two reverse primers: R1: 5’CTTGATCGGGAGCATAC3’ (complementary to reference allele; F+R1 amplicon length: 572 bp), and R2: 5’TGGAGCCAATTAATCACTTCAT3’ (complementary to deletion allele, F+R2 amplicon length: 707 bp). All three primers were used simultaneously in each single reaction. PCR was performed in a 6.25-μl reactions composed of 0.3 μl of 10 μM dilution of each primer (synthesized by Sigma-Aldrich), 0.125 μl of dNTPs mix (Promega, Madison, WI, USA), 0.05 μl of GoTaq DNA Polymerase (concentration 5 u/μl) (Promega), 1.25 μl of 5X colorless GoTaq reaction buffer (containing 7.5 mM MgCl_2_) (Promega), 2.925 μl of deionized water, and 1 μl of DNA (~30 ng). The following cycling conditions were used: 2 min at 95°C, followed by 35 cycles at 95°C for 20 sec, 56°C for 20 sec, and 72°C for 5 min, followed by 5 min at 72°C. The obtained PCR products were visualized on a standard 1.5% agarose gel.

### Statistical methods

The *APOBEC3B* deletion genotype-expression relationship was analyzed with the use of one-way analysis of variance (ANOVA) with post-test for linear trend, and t-test using Prism v. 4.0 (GraphPad, San Diego, CA).

HWE was assessed in the control groups using the chi-square test. Associations between the *APOBEC3B* deletion and breast or ovarian cancer risk were assessed using ORs and 95%CIs derived from logistic regression models. ORs(95%CIs) were estimated in the analyses assuming different models of inheritance, i.e., dominant (A3B+/− and A3B−/− vs. A3B+/+), recessive (A3B−/− vs. A3B+/− and A3B+/+), and additive (A3B−/− vs. A3B+/− vs. A3B+/+) models. Appropriate adjustments for the origin of the study and age (when data regarding age were available for particular case-control groups) were applied. Meta-analysis was performed with the use of Mantel-Haenszel method assuming dominant model of inheritance. MedCalc Statistical Software version 14.8.1 (MedCalc Software bvba, Ostend, Belgium; http://www.medcalc.org) was used for all logistic regression analyses and meta-analysis. All statistical tests were two-sided, and a p-value less than 0.05 was considered statistically significant.

## SUPPLEMENTARY MATERIALS FIGURES AND TABLES




