# Supplementary material for: The 30 kb deletion in the APOBEC3 cluster decreases APOBEC3A and APOBEC3B expression and creates a transcriptionally active hybrid gene but does not associate with breast cancer in the European population
Source: Oncotarget. 2017 Jul 19;8(44):76357–74. doi: 10.18632/oncotarget.19400 (PMC5652711; doi:10.18632/oncotarget.19400)
Supplement: Supplementary file 1 [file oncotarget-08-76357-s001.pdf]

The 30 kb deletion in the *APOBEC3* cluster decreases *APOBEC3A* and *APOBEC3B* expression and creates a transcriptionally active hybrid gene but does not associate with breast cancer in the European population

SUPPLEMENTARY MATERIALS

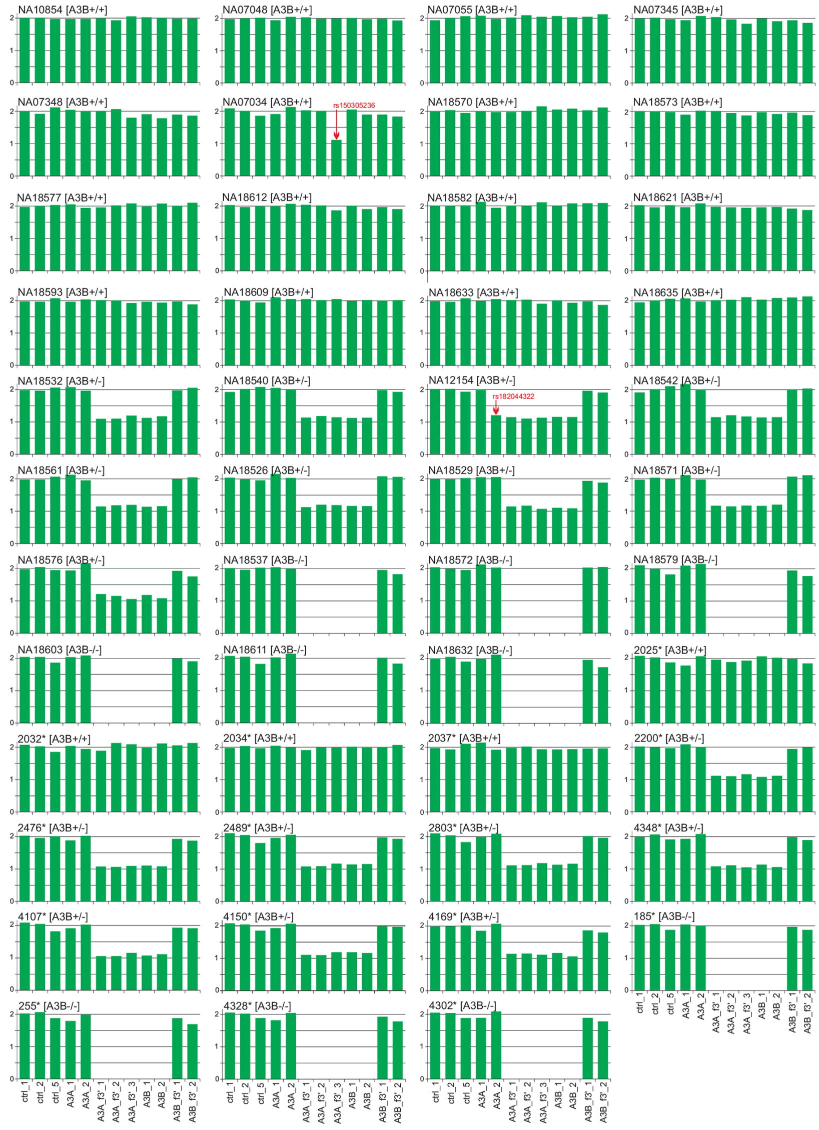

Supplementary Figure 1: Results of MLPA analyses of 31 samples from HapMap panel and 17 samples from women with breast and/or ovarian cancer (indicated with asterisks). The analysis was performed using the in-house designed A3Bdel_MLPA assay. The sample genotypes determined with the use of the A3Bdel_PCR assay are indicated in brackets. Bar plots represent normalized copy number values (y-axis) of each probe (x-axis). In two analyzed samples, i.e., NA07034 and NA12154, we observed an ~40% reduction of the individual probe signals (marked with red arrows). Sequencing analysis revealed that the reduction of signals in these samples resulted from rare SNPs (indicated in red) occurring in close proximity to the ligation sites of the respective MLPA probes.

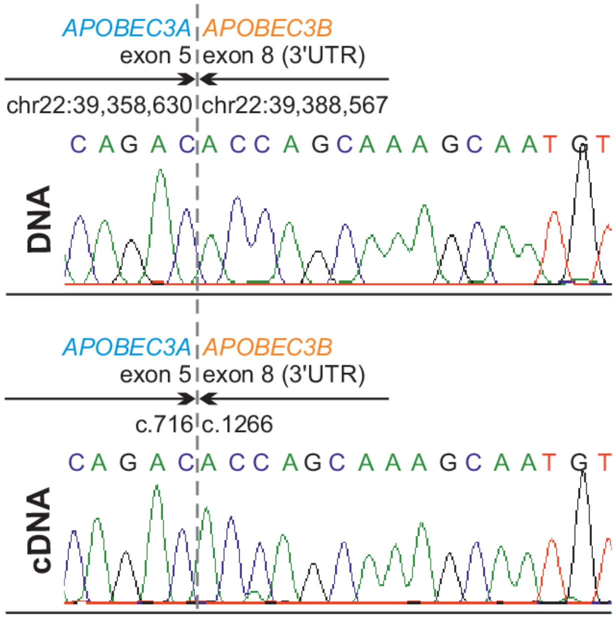

Supplementary Figure 2: Sanger sequencing of the deletion breakpoint (dotted line) in genomic DNA (upper panel) and cDNA (lower panel) confirms occurrence of the hybrid transcript in samples with the deletion; here, a sample with a homozygous A3B−/− deletion genotype.

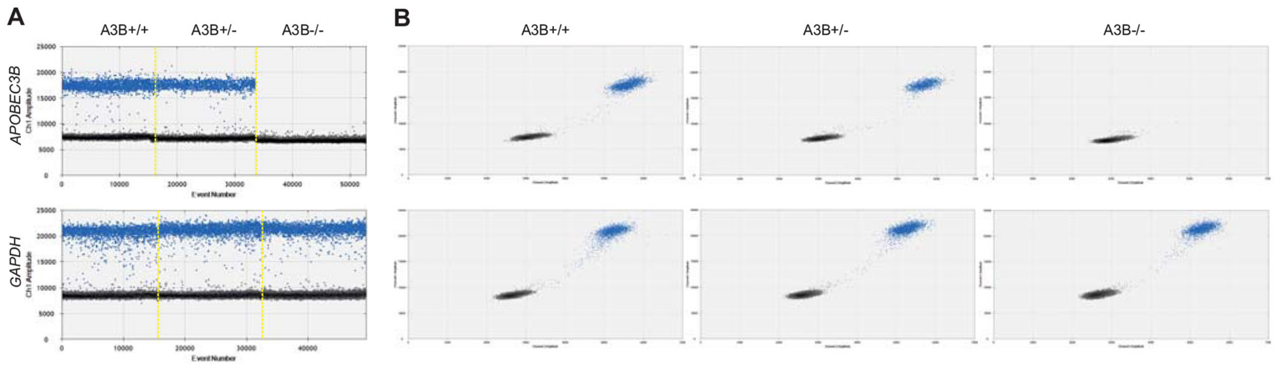

Supplementary Figure 3: Exemplary ddPCR results of expression (transcript) level analysis of *APOBEC3B* and reference *GAPDH* in three representative HapMap samples with the A3B+/+, A3B+/−, and A3B−/− genotypes. Note that to obtain comparable numbers of positive signals (droplets), prior to the *GAPDH* analysis, the samples were additionally 400x diluted. The ddPCR analysis and data visualization was performed with the use of BIO-RAD QuantaSoft. The expression level (number of cDNA copies of particular gene in analyzed sample) is proportional to the number of positive droplets. Positive and negative droplets are marked in blue and black, respectively. **(A)** Visualizations in 1D Amplitude view; y-axis – channel 1 amplitude (fluorescent signal intensity), x-axis – sample with the particular genotype (indicated above). **(B)** Visualizations in 2D Amplitude view (corresponding to panel A); y-axis – channel 1 amplitude; x-axis – channel 2 amplitude.

Supplementary Table 1: Detailed characteristics and sequences of the probes included in the A3Bdel_MLPA assay.[a]

See Supplementary File 1

Supplementary Table 2: Analysis of the association of the *APOBEC3B* deletion with breast cancer risk using different models of inheritance

Group	Model of inheritance	OR(95%CI)	Adjusted OR(95%CI)
GDANSK BC cases (n=523) vs. unselected controls (n=853)	dominant A3B+/− and A3B−/− vs. A3B+/+	1.31(0.94-1.82) p=0.11	-
	recessive* A3B−/− vs. A3B+/− and A3B+/+	1.09(0.18-6.53) p=0.93	-
	additive A3B−/− vs. A3B+/− vs. A3B+/+	1.28(0.94-1.75) p=0.12	-
SZCZECIN BC cases (n=2009) vs. unselected controls (n=2005)	dominant A3B+/− and A3B−/− vs. A3B+/+	0.89(0.74-1.07) p=0.20	0.90(0.75-1.08)[a] p=0.26
	recessive* A3B−/− vs. A3B+/− and A3B+/+	2.00(0.68-5.87) p=0.21	2.14(0.73-6.3)[a] p=0.17
	additive A3B−/− vs. A3B+/− vs. A3B+/+	0.91(0.76-1.09) p=0.31	0.93(0.78-1.11)[a] p=0.41
SZCZECIN BC cases (n=2005) vs. NH-controls (n=615)	dominant A3B+/− and A3B−/− vs. A3B+/+	1.03(0.78-1.36) p=0.83	0.95(0.70-1.29)[a] p=0.73
	recessive* A3B−/− vs. A3B+/− and A3B+/+	0.61(0.21-1.79) p=0.37	0.34(0.10-1.13)[a] p=0.08
	additive A3B−/− vs. A3B+/− vs. A3B+/+	1.00(0.77-1.30) p=1.00	0.90(0.68-1.21)[a] p=0.49
GDANSK+SZCZECIN BC cases (n=2532) vs. unselected controls (n=2858)	dominant A3B+/− and A3B−/− vs. A3B+/+	0.98(0.83-1.15) p=0.79	0.97(0.83-1.14)[b] p=0.73
	recessive* A3B−/− vs. A3B+/− and A3B+/+	1.70(0.69-4.16) p=0.25	1.70(0.69-4.19)[b] p=0.25)
	additive A3B−/− vs. A3B+/− vs. A3B+/+	1.0(0.85-1.16) p=0.96	0.99(0.85-1.16)[b] p=0.90
GDANSK+SZCZECIN familial BC cases (n=640) vs. unselected controls (n=2858)	dominant A3B+/− and A3B−/− vs. A3B+/+	1.06(0.82-1.36) p=0.67	1.15(0.87-1.52)[b] p=0.32
	recessive* A3B−/− vs. A3B+/− and A3B+/+	1.12(0.24-5.27) p=0.89	0.88(0.17-4.71)[b] p=0,88
	additive A3B−/− vs. A3B+/− vs. A3B+/+	1.06(0.83-1.35) p=0.66	1.14(0.87-1.48)[b] p=0.36

[a]adjusted for age; [b]adjusted for the origin of the study; *in the recessive model we obtained relatively low statistical power due to the low frequency of homozygous deletions.

Supplementary Table 3: Analysis of the association of the *APOBEC3B* deletion with ovarian cancer risk using different models of inheritance

Group	Model of inheritance	OR(95%CI)	Adjusted OR(95%CI)
GDANSK<br>OC cases (n=343) vs. unselected controls (n=853)	dominant<br>A3B+/− and A3B−/− vs. A3B+/+	0.77(0.50-1.19)<br>p=0.24	-
	recessive*<br>A3B−/− vs. A3B+/− and A3B+/+	1.66(0.28-9.99)<br>p=0.58	-
	additive<br>A3B−/− vs. A3B+/− vs. A3B+/+	0.82(0.54-1.22)<br>p=0.32	-
VILNIUS<br>OC cases (n=97) vs. unselected controls (n=209)	dominant<br>A3B+/− and A3B−/− vs. A3B+/+	0.66(0.27-1.61)<br>p=0.36	-
	additive<br>A3B−/− vs. A3B+/− vs. A3B+/+	0.78(0.34-1.77)<br>p=0.55	-
GDANSK + VILNIUS<br>OC cases (n=440) vs. unselected controls (n=1062)	dominant<br>A3B+/− and A3B−/− vs. A3B+/+	0.75(0.51-1.10)<br>p=0.14	0.75(0.51-1.11)[b]<br>p=0.15
	recessive*<br>A3B−/− vs. A3B+/− and A3B+/+	2.42(0.49-12.05)<br>p=0.28	2.44(0.49-12.14)[b]<br>p=0.28
	additive<br>A3B−/− vs. A3B+/− vs. A3B+/+	0.81(0.56-1.16)<br>p=0.25	0.81(0.56-1.16)[b]<br>p=0.25

[b]adjusted for the origin of the study; *in the recessive model we obtained relatively low statistical power due to the low frequency of homozygous deletion, the recessive model was not tested in the VILNIUS group due to lack of homozygous deletions in the control group.
